# Composition and Quantitation of Microalgal Lipids by ERETIC ^1^H NMR Method

**DOI:** 10.3390/md11103742

**Published:** 2013-09-30

**Authors:** Genoveffa Nuzzo, Carmela Gallo, Giuliana d’Ippolito, Adele Cutignano, Angela Sardo, Angelo Fontana

**Affiliations:** Institute of Biomolecular Chemistry, National Research Council, Via Campi Flegrei 34, Pozzuoli 80078, Naples, Italy

**Keywords:** microalgae, biofuel, functional products, fatty acid, lipid

## Abstract

Accurate characterization of biomass constituents is a crucial aspect of research in the biotechnological application of natural products. Here we report an efficient, fast and reproducible method for the identification and quantitation of fatty acids and complex lipids (triacylglycerols, glycolipids, phospholipids) in microalgae under investigation for the development of functional health products (probiotics, food ingredients, drugs, *etc.*) or third generation biofuels. The procedure consists of extraction of the biological matrix by modified Folch method and direct analysis of the resulting material by proton nuclear magnetic resonance (^1^H NMR). The protocol uses a reference electronic signal as external standard (ERETIC method) and allows assessment of total lipid content, saturation degree and class distribution in both high throughput screening of algal collection and metabolic analysis during genetic or culturing studies. As proof of concept, the methodology was applied to the analysis of three microalgal species (*Thalassiosira weissflogii*, *Cyclotella cryptica* and *Nannochloropsis salina*) which drastically differ for the qualitative and quantitative composition of their fatty acid-based lipids.

## 1. Introduction

Microalgae are a recognized source of fatty acids (FA) and fatty acid-based lipids of potential interest in preparation of functional health products [1,2]. Unlike terrestrial crops, these photoautotrophic microoganisms can directly produce polyunsaturated fatty acids (PUFA) and, although microalgae are not suitable for direct human consumption, their nutritional value can also be exploited if added to animal feeds [3,4,5,6,7,8]. In recent years, the interest in microalgal fatty acids has increased as a consequence of the consideration that they can be a suitable source of third generation biofuels such as crude oil (triacylglycerols) or after conversion to methyl esters (biodiesel) [9,10].

Composition of algal lipids greatly depends on genetic and phenotypic factors, including environmental and culture conditions [10,11,12]. Thus, analytical tools for their assessment have become rapidly important in comparative and screening studies [13]. Conventional protocols for analysis of lipids typically involve solvent extraction and chromatographic analysis [14,15,16,17,18]. These techniques are time consuming and labor-intensive, which limits their application to quickly assay a large number of samples [13]. To address this point, a fluorescent method based on Nile Red (9-diethylamino-5*H*-benzo[α]phenoxazin-5-one) has been recently introduced for assessment of neutral lipids [19,20,21]. However, the lipid-soluble fluorescent probe requires daily calibration and has a number of technical disadvantages including specificity of cell response and nonlinear emission. More recently, spectroscopic methods including proton and carbon nuclear magnetic resonance (^1^H and ^13^C NMR) have also been reported to monitor the transesterification reaction and formation of by-products during biodiesel production [22,23,24,25,26,27,28] but, to the best of our knowledge, they have never been applied to screen algal lipids.

In this study, we discuss a simple procedure based on ^1^H NMR spectroscopy for identification and quantitation of different species of lipids in few milligrams of rough microalgal extracts. The method offers a rapid assessment of content and composition of major lipid classes, including triacylglycerols (TAG), phospholipids (PL), glycolipids (GL) and free fatty acid (FFA), which permits the routine analysis of a large number of samples.

## 2. Results and Discussion

### 2.1. Sample Preparation

**Figure 1:**
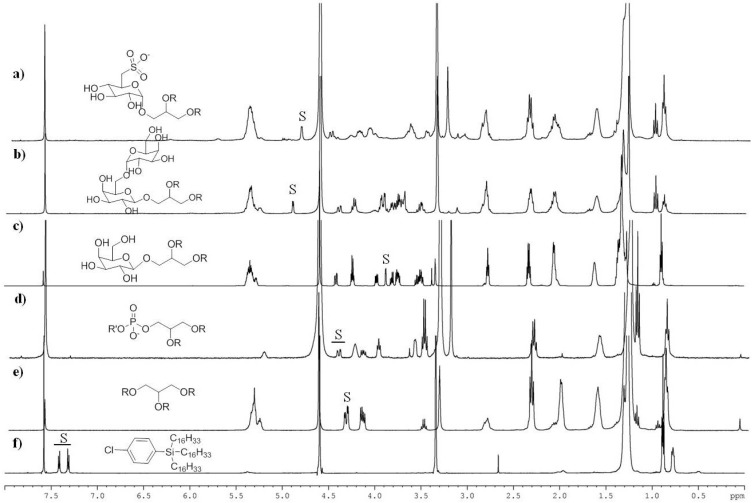
^1^H NMR spectra (CDCl_3_:CD_3_OD 1:1) of pure lipid compounds and surrogate standard (CPHS). Diagnostic signals (*S*) that allow recognition and quantitation of each lipid class are listed in Table 1. R = fatty acyl residue. (**a**) sulfoquinovosyldiacylglycerols; (**b**) digalactosyldiacylglycerolstriacylglycerols; (**c**) monogalactosyldiacylglycerols; (**d**) phospholipids; (**e**) triacylglycerols; (**f**) CPHS (surrogate standard).

After harvesting by centrifugation, cell pellets were washed twice by 0.5 M ammonium formate to remove salt and lyophilized [29,30,31]. Dried samples were weighted and directly extracted by the modified Folch method [32] after addition of the internal standard (20 µg per mg of dry sample) to measure extraction accuracy. Different compounds were tested as surrogate internal standards (data not shown) but the best results were obtained with 4-chlorophenyl-trihexadecylsilane (here abbreviated as CPHS) that showed stability under the analytical conditions and lipophilic properties similar to those of fatty acid-based lipids. Furthermore, CPHS was featured by ^1^H-NMR signals in regions different from those of interest for the characterization of the major classes of microalgal lipids (Figure 1). A stock solution of the surrogate standard was prepared by dissolving 50 mg of product in 5 mL of THF (10:1 w/v). The solution was stable for several months at 0 °C. To evaluate the recovery, scalar concentrations of CPHS ranging from 0.1 to 1 mg were spiked to artificial mixtures composed of PL, TAG and proteins (ovalbumin). The results showed a very satisfactory recovery of the surrogate standard (89.6 ± 4.2 percent, *n* = 5), as measured by the NMR protocol used for the lipid analysis. Alternatively to the modified Folch method, we also tested a simplified protocol of extraction that does not require partition of the organic material against water. To this aim, after washing with ammoniate formate, the lyophilized algal pellet was spiked with 20 µg of CPHS for each mg of dry pellet and extracted by sequential addition of 5 mL MeOH and 15 mL of CHCl_3_:CH_3_OH (2:1 v/v). The insoluble material was eliminated by centrifugation and the supernatant was directly dried at reduced pressure.

### 2.2. NMR Methodology

To facilitate the assignment of peaks diagnostic for each lipid class, proton NMR spectra of standard compounds were recorded under the same conditions used for the analysis of the microalgal extracts (Figure 1). As expected in consideration of the chemical similarities, the inspection of the NMR spectra showed severe peak overlapping, especially in the regions of aliphatic (below 2 ppm) and olefinic (above 5 ppm) protons. Nevertheless, each class of molecules showed diagnostic signals that could be selected as NMR markers with no or only limited interference by other compounds (Table 1). The unambiguous identification of these signals, as well as their characterization in microalgal extracts (see below), was also supported by a complete set of 2D NMR experiments, including COSY, HSQC and HMBC spectra (not shown).

**Table 1:** Assignment of ^1^H NMR diagnostic signals (*S*) for the identification and assessment of the major lipid classes in microalgae. TAG = triacylglycerols; PL = phospholipids; GL= glycolipids; MGDG = monogalactosyldiacylglycerols; DGDG = digalactosyldiacylglycerols; SQDG = sulfoquinovosyldiacylglycerols; TFA= total fatty acids; UFA = unsaturated fatty acids.

*S*	*Chemical Shift* (ppm)	*n*	Chemical Assignment	Lipid Class
**1**	4.34	2	methylene protons of glycerol	TAG
**2**	4.53–4.38	1	methylene protons of glycerol	PL + GL
**3**	3.88	1	methine proton at C4 of galactose	MGDG
**4**	4.90	1	anomeric proton of galactose	DGDG
**5**	4.80	1	anomeric proton of sulfoquinovoside	SQDG
**6**	2.35 ^a^	2	methylene protons α to carboxy group	TFA
**7**	2.06 ^b^	4	allylic protons	UFA

^a ^large signal between 2.39 and 2.28 ppm; ^b^ large signal between 2.15 and 1.98 ppm.

Product quantitation was accomplished by ERETIC method that provides a reference electronic signal as external standard [33]. The reference signal is an intrinsic property of the spectrum and can be accurately calibrated against an external compound of known concentration. The properties of the electronic signal allow the extension of calibration intervals that usually require adjustments every one or two months. In this study, the ERETIC peak was calibrated on the NMR doublet at 7.40 ppm (*J* = 8.0 Hz) of 2.20 µmol of CPHS in 700 µL CDCl_3_/CD_3_OD 1:1 (v/v). Concentrations of FA and complex lipids were determined in microalgal extracts according to the equation (*1*):

C = k A_S_ n^−1^ (*1*)

where C is the concentration of the analyte in mole, n is the number of protons (one for CH; two for CH_2_; three for CH_3_), k is the ratio between the concentration and peak area of the ERETIC signal normalized to a single hydrogen, and A_S_ is the area of the NMR signal *S* diagnostic for the lipid class of interest (Table 1). To minimize the effect due to signal saturation and relaxation delay, ^1^H NMR experiments were recorded with one scan using a 90° pulse. Under these conditions, the quantitative measurement is accurate with a relative error below 5% for every lipid class considered in this study.

The NMR method was tested on three algal species, namely *Thalassiosira weissflogii* (ICBCP09), *Nannochloropsis salina* (CCMP369) and *Cyclotella cryptica* (CCMP331), which were cultured in 1 L carboy and harvested in stationary phase.

**Figure 2:**
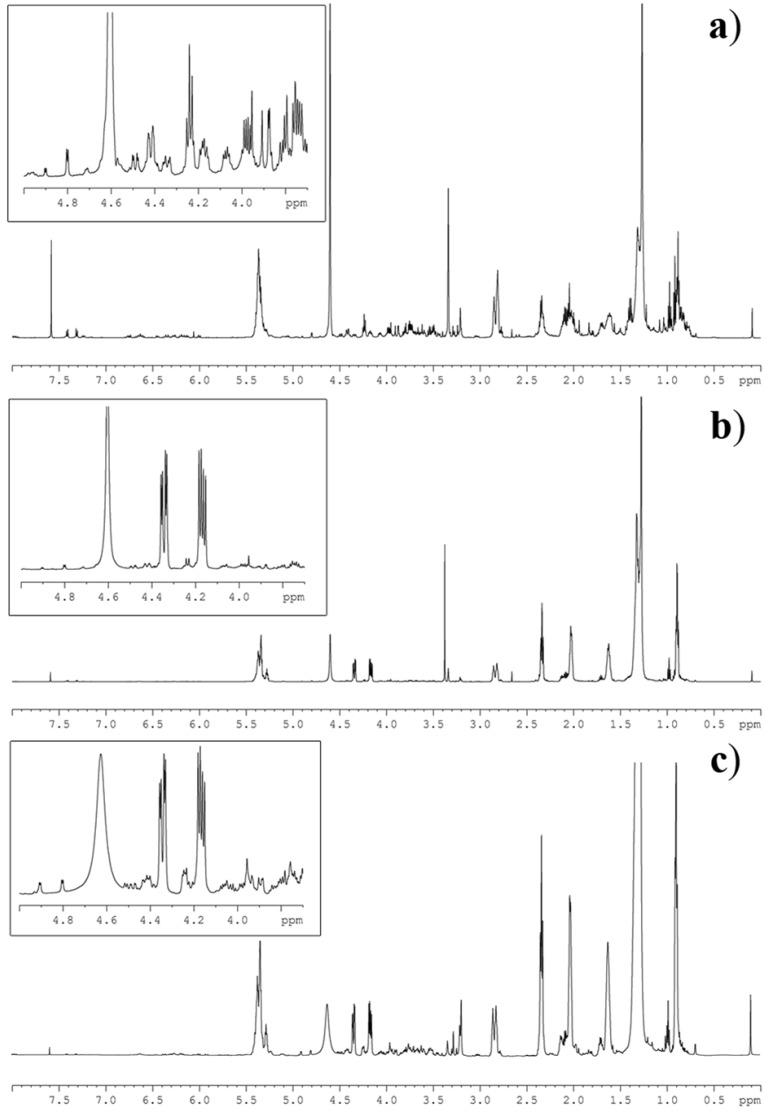
^1^H NMR spectrum of lipid extracts of the microalgae (**a**) *Thalassiosira weissflogii* (Bacillariophiceae), (**b**) *Cyclotella cryptica* (Bacillariophiceae) and (**c**) *Nannochloropsis salina* (Eustigmatophyceae). The inserts show magnification of the NMR region containing the diagnostic signals as identified in Table 1.

As shown in Figure 2, ^1^H NMR spectra of the microalgal extracts were featured by a complex profile showing severe peak overlapping of signals due to triacylglycerols (TAG), free fatty acids (FFA), phospholipids (PL) and glycolipids (GL). This last group was further composed of monogalactosyldiacylglycerols (MGDG), digalactosyldiacylglycerols (DGDG) and sulfoquinovosyldiacylglycerols (SQDG). Regardless of this complexity, the diagnostic signals (*S*) of each group were clearly discernible and could be integrated by routine Bruker software. Area determination was established by the average on three different measurements (Table 2). In particular, concentration of TAG was ascertained on the signal at δ 4.34 (dd, *J* = 4.0, 12.0 Hz) of the primary glycerol protons, whereas the three classes of glycolipids were determined by the sugar resonances at δ 3.88 (d, *J* = 3.0 Hz) of H-4 of MGDG, and at δ 4.90 (d, *J* = 3.5 Hz) and 4.80 (d, *J* = 3.7 Hz) of the anomeric protons of DGDG and SQDG, respectively.

**Table 2:** Qualitative and quantitative (in µmol/g of extract) analysis of lipid extracts of *T. weissflogii* (TW), *N. salina* (NS) and *C. cryptica* (CYC) based on the area (A_S_) of the diagnostic signals (*S*). Abbreviations are in agreement with Table 1.

	Integrated Signal	TW		NS		CYC	
		A_S_	C (µmol)	A_S_	C (µmol)	A_S_	C (µmol)
TAG	1	0.198	23.0	27.43	140.0	52.04	530.0
PL + GL	2	1.055	192.4	6.913	22.2	3.139	79.8
MGDG	3	0.277	75.0	0.946	6.4	0.726	28.4
DGDG	4	0.033	40.0	1.087	4.6	0.757	18.0
SQDG	5	0.184	9.2	1.113	5.6	0.229	7.0
TFA	6	2.642	453.8	109.8	464.4	166.7	1749.6
UFA	7	5.083	436.5	149.5	316.3	176.8	927.7

Phospholipids could not be measured directly because of the partial overlapping of their signals with those of other lipid components. Nevertheless, their percentage was simply inferred according to the equation (*2*) by difference between pooled concentrations of PL and GL, as assessed on the signal S_2_ in the region between 4.53 and 4.38 ppm (*sn*-1 glycerol protons), and the concentration of GL as previously established by A_S3_, A_S4 _and A_S5_ (Table 1).


C_PL_ = k [(A_S2_ − (A_S3_ + A_S4_ + A_S5_)] (*2*)

The integrated value of the methylene protons centered at δ 2.35 ppm was used as determinant indicative of total fatty acid (TFA) moles in the algal extract. This value corrected by contribution of fatty acids bound to complex lipids, namely PL, TAG and GL, gave the simple relation (*3*) to calculate the moles of free fatty acids (FFA) in the sample.


[FFA] = [TFA] − (3[TAG] + 2[PL] + 2[GL])
(*3*)

Finally, in agreement with Annarao *et al.* [34], levels of saturated fatty acids (SFA) and unsaturated fatty acids (UFA) were established by the area of allylic (A_S7_) and α-methylene protons (A_S6_), according to equations (*4*) and (*5*).


C_UFA_ = k A_S7_/4 (*4*)

CSFA = k [(AS6/2) − (AS7/4)]
(*5*)

### 2.3. Lipid NMR Analysis

**Figure 3:**
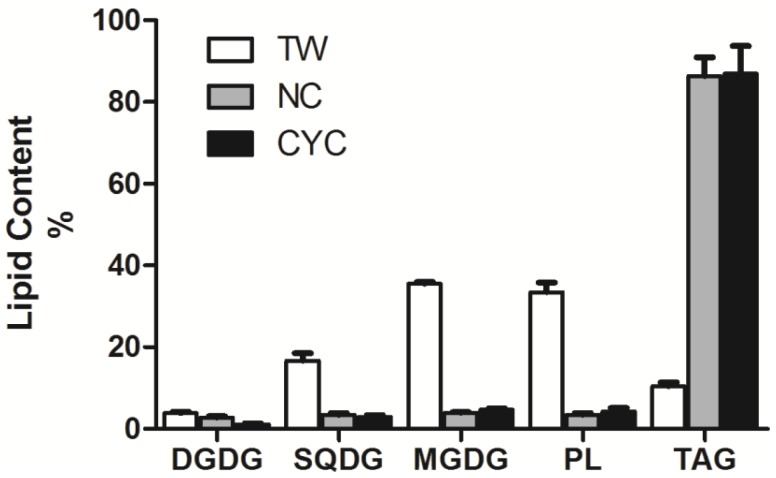
Content percent of the major classes of complex lipids in the three strains of marine microalgae *T. weissflogii* (TW), *N. salina* (NS) and *C. cryptica* (CYC). Data indicate mean value and standard deviation on three biological replicates. Lipid abbreviations are in agreement with Table 1.

A detailed protocol for preparation and NMR analysis of fatty acid-based lipids in microalgal samples of *T. weissflogii* (TW), *N. salina* (NS), *C. cryptica* (CYC) is reported in the Experimental Section. The three species belong to different taxonomic classes and therefore show different lipid compositions [35,36,37]. Lipid quantitation (µmol/mg dry pellet) per each microalga is shown in Table 3, whereas percent composition of the major lipid classes is reported in Figure 3. Data are related to replicates of three different cultures of each algal strain. For absolute assessment (lipid weight per cell unit or per unit of dry pellet), one simply needs to take into account the average molecular weight of each class of lipid. It is worth noting that only a few milligrams (~10 mg) of dry pellet were necessary to complete the analysis regardless of the specific lipid composition of each strain. In fact, the methodology proved to be suitable for analysis of samples like TW, that were mostly rich in polar lipids (GL and PL accounted for 80% of total lipids), or NS and CYC that show a predominant presence of triglycerides (60%–80% of total lipids). Significantly, the method succeeded in determining lipid families (*i.e.*, DGDG) that were either far below 1 µmol per mg of total lipids (*i.e.*, see lipid analysis of NS) or below 3% of total lipid extract (Figure 3), allowing us to obtain qualitative and quantitative information on minor components of the extract that would not be otherwise measurable.

**Table 3:** Quantitative analysis (µmol/mg of dry pellet) of lipids in *T. weissflogii* (TW), *N. salina* (NS) and *C. cryptica* (CYC). Abbreviations are in agreement with Table 1. Data indicate mean value and standard deviation on three biological replicates.

	SQDG	DGDG	MGDG	PL	TAG	FFA	UFA	SFA
**TW**	9.2 ± 1.4	40.0 ± 9.0	75.0 ± 9.0	68.2 ± 16.8	23.0 ± 1.6	35.6 ± 15.0	436.5 ± 32.7	17.3 ± 4.9
**NS**	5.6 ± 0.8	4.6 ± 1.4	6.4 ± 0.8	5.6 ± 0.8	141.8 ± 22.0	55.4 ± 5.4	316.3 ± 44.0	148.1 ± 24.8
**CYC**	7.0 ± 3.4	18.0·± 5.6	28.4 ± 4.8	26.4 ± 11.0	530.0 ± 93.0	147.6 ± 29.4	927.7 ± 121.2	821.9 ± 130.2

Full analysis of microalgal sample required only few minutes and NMR assessment can be fully automated through the use of simple software (not shown). Although this is beyond the aim of this work, in view of introducing a further simplification of the method, we evaluated extraction of the samples without partition against water of the CHCl_3_/CH_3_OH phase. Chemical shift and resolution did not differ from samples obtained by the modified Folch method, even if the spectra of algae extracted by the simplified procedure showed an additional signal between 4.50 and 4.38 ppm due to an uncharacterized hydrophilic component. The presence of this material hampered the full application of the NMR methodology since the NMR signal covered the diagnostic resonances of PL and GL. Nevertheless, the NMR spectra of these samples retained the capacity to provide information on TFA, TAG, FFA, UFA and SFA, thus suggesting the potential use of the simplified procedure in analysis that are focused only on these lipid classes (*i.e.*, crude oil screening or biodiesel synthesis).

## 3. Experimental Section

### 3.1. General

All the chemicals and solvents (Sigma-Aldrich, Milan, Italy) were of analytical reagent grade and were used without any further purification. Standards were of commercial origin (Sigma-Aldrich, Milan, Italy), only glycolipids were purified from natural source [38] or synthesized according to Manzo *et al.* [39,40,41]. Microalgae were harvested by centrifugation on Allegra X-12R centrifuge (Beckman Coulter, Fullerton, CA, USA) and lyophilized in a Savan Micro Modulyo freeze dryer (Thermo Scientific, Austin, TX, USA). ^1^H NMR spectra were recorded on Bruker DRX 600 spectrometer equipped with an inverse TCI CryoProbe. Peak integration, Eretic measurements and spectrum calibration were obtained by the specific subroutines of Bruker Top-Spin 3.1 program. Spectra were acquired with 14 ppm of spectral width (8417.5 Hz), 32 K of time domain data points, 90° pulse, 32 K spectrum size, and processed with 0.6 Hz of line broadening for the exponential decay function.

### 3.2. Algal Culturing

*Thalassiosira weissflogii* (ICBCP09) is an original isolate obtained from Stazione Zoologica “A. Dohrn” (Naples, Italy). *Nannochloropsis salina* (CCMP369) and *Cyclotella cryptica* (CCMP331) were purchased from Bigelow Laboratories. Microalgae were cultured at 20 ± 2 °C in f/2 medium [42] by 1 L carboy at 100 μmol m^−2^ s^−1^ with 14:10 h (light:dark) photoperiod. Cells were harvested by centrifugation at 3750 rpm for 10 minutes at 4 °C.

### 3.3. Lipid Extraction

Cells were washed by 0.5 M ammonium formate solution, frozen in liquid nitrogen and lyophilized. 4-chlorophenyl-trihexadecylsilane (20 µg/mg dry pellet) was added to each sample as internal standard. Unless otherwise specified, microalgal extracts were prepared according to the modified Folch method [32]. Organic layer was recovered by centrifugation (3750 rpm for 5 min) and dried at reduced pressure. Extractions were repeated three times.

### 3.4. NMR Analysis

Crude microalgal extracts were dissolved in 700 µL CDCl_3_/CD_3_OD 1:1 (v/v) and transferred to the 5-mm NMR tube. Chemical shift was referred to CHD_2_OD signal at δ 3.34. Quantitative assessment was established by the ERETIC method in agreement with reference 33. The ERETIC signal was calibrated on the doublet signal at δ 7.40 of 4-chlorophenyl-trihexadecylsilane (2.20 µmol in 700 µL CDCl_3_/CD_3_OD 1:1).

### 3.5. Protocol for General Analysis of Fatty Acid-Based Lipids in Microalgae

Microalgal samples should be placed in glass tubes with Teflon-lined screw caps and frozen in liquid nitrogen immediately after washing.

1. Lyophilize frozen sample.
2. Cover dry sample with 5 mL methanol and kept at 4 °C for 1 min.
3. Add 20 µg (24.4 × 10^−3^ µmol) internal standard [(4-chlorophenyl)-trihexadecylsilane] for each mg of dry sample.
4. Add 10 mL chloroform.
5. Homogenized and incubate with shaking for 5 min.
6. Centrifuge (3750 rpm) for 5 min at room temperature.
7. Transfer supernatant to a fresh tube.
8. Suspend solid residue in 15 mL chloroform:methanol (2:1 v/v) and incubate with shaking for 2 min.
9. Repeat steps 6 and 7.
10. To combined supernatants from steps 7 and 9, add 7 mL deionized water.
11. Vortex, centrifuge and discard the upper phase.
12. Recover lower phase (organic extract) with a Pasteur pipette and transfer to a glass rotary evaporator flask.
13. Remove solvent and dry sample under vacuum at room temperature.
14. Dissolve dry extract in 700 µL CD_3_OD/CDCl_3_ (1:1 v/v).
15. Transfer to a fresh NMR tube.
16. Acquire ^1^H NMR spectrum (ns = 1, ds = 0, spectral with 14 ppm, O1P = 5).
17. Integrate diagnostic signal (Figure 2) with the “Process—Integrate” function of the Bruker Top Spin software or corresponding programs of other manufacturers.
18. Calculate concentration (µmol/L) of each lipid class by the ERETIC function of the same software.

In order to reduce salt content in samples of marine algae, it is strongly advised to wash the cell pellet after harvesting. For 1 g wet weight, rinsing protocol is as follows:

- suspend algal pellet in 5 mL of 0.5 M ammonium formate solution.
- centrifuge the sample (3600 rpm) for 5 min at room temperature.
- remove the supernatant.
- repeat twice the above steps.

After NMR assessment, lipid extracts may be concentrated under a stream of nitrogen for chromatographic analysis (TLC, HPLC, LCMS) and preparation of derivatives. For minimum oxidation, all extraction solvents should contain 0.01% BHT.

## 4. Conclusions

Lipid assessment has become crucial in many studies focused on biotechnological use of microalgae. The present method offers a fast and simple analysis based on single-scan NMR spectroscopy. Without purification and manipulation, the procedure allowed us to identify and quantify the major lipid classes, as well as to establish the levels of free fatty acids or saturated and unsaturated components in the organic extracts. As proved with three different microalgal species, the methodology is suitable for the analysis of samples with homogeneous or heterogeneous lipid composition. The potential of 600 MHz spectrometer permits identification and quantitation of single components in complex mixtures; however, more routinely, instruments (400 and 500 MHz) are also suitable for application during high-throughput screening of algal collection or metabolic analysis in genetic and culturing studies. Other NMR methods based on detection of ^13^C and ^31^P nuclei can allow resolution of specific lipid families, such as acylglycerols [27] or phospholipids [43,44]. Although these techniques were not considered in the present study, they can be easily integrated in the method. Furthermore, NMR is a nondestructive technique, thus, the lipid extracts can be recovered and used for further investigations with other analytical tools, including LCMS and GCMS. The use of the ERETIC approach, which has already been proposed for assessing metabolite concentrations *in vivo* [33], requires monthly calibration, thus leading to a further advantage in the processing of a large number of samples.
